# Feasibility of Using Dynamic Appraisal of Situational Aggression to Reduce Agitation in an Inpatient Psychiatric Unit

**DOI:** 10.7759/cureus.96243

**Published:** 2025-11-06

**Authors:** Diane E Mathew, David P Mathews, Dongliang Wang, Derek W Empey, Elena C Nichita, Luba Leontieva

**Affiliations:** 1 Psychiatry, State University of New York Upstate Medical University, Syracuse, USA; 2 Public Health and Preventive Medicine, State University of New York Upstate Medical University, Syracuse, USA; 3 Pharmacy, State University of New York Upstate Medical University, Syracuse, USA; 4 Psychiatry, State University of New York Upstate Medical University, Syracuse, USA

**Keywords:** agitation, nursing, prediction tools, quality improvement research, safety

## Abstract

Agitation in inpatient psychiatric units is common and is an important area for research given its impact on staff and patients. This study explores the feasibility and utility of the Dynamic Appraisal of Situational Aggression (DASA) risk assessment instrument. The quality improvement project was implemented on two acute care inpatient psychiatric units in upstate New York (NY) over a two-and-a-half-month period. The study investigated the association of DASA scores with interventions. The interventions included emergent medication for agitation, seclusion, or restraint as measures of agitation. The study compared DASA scoring related to agitation levels in patients with the occurrence of administration of emergent medication, seclusion, or restraints for the same patients. This was done to investigate whether a higher DASA score correlated with more interventions. A two-sample t test was used to explore the link between DASA scores and interventions, including emergent medication, seclusion, and restraint. The study also investigated mental health nursing perspectives of the scoring tool through descriptive analysis. An anonymous survey was administered to nurses with five questions designed to assess how easy the scoring tool was to use and their perception of its utility. Analysis showed no association between the DASA score and interventions for agitation. The feasibility of the DASA tool was achieved, as survey results showed that nurses found DASA user-friendly. Potential reasons for these results could be associated with multiple factors, including the nature of agitation on these inpatient units and barriers to compliance with the tool.

## Introduction

Aggression, also labeled as agitation, in inpatient psychiatric units is important to investigate, as it has an impact on both staff and patients. Agitation on inpatient units leads to physical and psychological consequences for mental health staff and the overall organization. It is associated with increased anger and fear, higher turnover, decreased morale, and more frequent medical errors [1]. There have been interventions such as Project BETA, a project in the Emergency Department (ED), that systematized the utilization of best evidence and consensus-based recommendations. This intervention utilized verbal de-escalation as a first-line technique and pharmacotherapy to treat the most likely underlying etiology of agitation, aiming to manage agitation events in the hospital that may place both patients and staff at risk [2].

Though there are efforts to find methods to manage agitation, a key component to consider is how to quickly assess and determine the potential for agitation before unsafe events occur. In a study analyzing aggression on inpatient units, the utility of static and dynamic factors in the assessment of violence was emphasized, along with the classification of aggression type (impulsive/reactive, organized/predatory/instrumental, psychotic) [3]. Another study of aggression in psychiatric facilities showed that impulsive aggression occurs most often, about 58% of the time [4]. The Dynamic Appraisal of Situational Aggression (DASA) scoring scale is a nursing assessment tool used globally to assess agitation. It has been applied in multiple settings, including the ED, with predictive value for agitation [5].

In inpatient psychiatric units, studies have shown the validity of this tool in predicting agitation in specific populations. On forensic mental health units across multiple regions of the world, such as Australia, the UK, and China, the DASA scoring tool has shown predictive validity [6-8]. A systematic review in 2020 assessing structured schemas to predict imminent aggression explored the predictive value of the DASA scoring tool. It was observed that in the studies evaluated, the definition of aggression varied (all aggression versus physical aggression), but eight studies reported information on physical aggression and five on verbal aggression. There was a large pooled effect size for any aggression and physical aggression against people (Hedges' g = 0.88; 95% CI, 0.62-1.15) [9]. A study in Finland found an association between DASA scores and interventions deployed. For 64 patients with high DASA observations, 91.2% of occurrences of high agitation led to the use of an intervention, most commonly as-needed medication, seclusion, or focused discussion with nursing staff [10]. However, a study conducted in England in 2009 on a forensic unit with individuals with severe personality disorders showed no significant validity for the DASA scale [11].

Furthering research on the relationship between DASA scores and the interventions used will allow for a better understanding of this scoring tool’s efficacy across different populations. The goal of this study is to assess, through a quality improvement project, the feasibility of using the DASA score in a population that has not been previously studied. This research, conducted in upstate New York, is original in nature, as there are limited studies using this scoring tool in the United States and a need for further research in units that are not forensic-based. The study described below addresses this research gap.

## Materials and methods

The State University of New York Upstate Institutional Review Board (IRB) reviewed this retrospective case-control study and approved it as exempt from further review. The project involved secondary research for which consent was not required. The information was gathered in a manner in which the human subjects' identities could not be readily ascertained directly or through identifiers linked to the subjects, and the investigators did not contact the subjects or reidentify them. The study was conducted in accordance with all applicable State University of New York (SUNY) Upstate Medical University human subject research requirements as well as applicable federal regulations.

This study was conducted in upstate New York on two acute inpatient psychiatric units. As part of a departmental quality improvement initiative (i.e., not for the purposes of this study), nursing staff on both psychiatric units were given training during multiple sessions held during nursing huddles on how to properly score using DASA. All nurses on the units were trained, totaling more than 20 nurses. The nurses recorded these values on paper logs over a two-and-a-half-month period for each patient who was on the unit. Nurses were instructed to complete one DASA assessment for each of their patients during their eight- to twelve-hour shift, as nurses changed shifts and could have different reports of patient behavior, though scoring often occurred only once per day.

The DASA scoring tool has seven categories to be scored based on the past 24 hours. The categories are irritability, impulsivity, unwillingness, sensitivity to perceived provocation, being easily angered when requests are denied, negative attitudes, and verbal threats. The patient is given a score of 1 in the category if the behavior described is increased, and 0 if the behavior remains at baseline. A total score out of seven is calculated from the above categories. Risk categories are listed as low for scores of 0-1, moderate for 2-3, and high for 4-7. Another section on the DASA records the presence of physical or verbal aggression against objects, patients, or staff by placing an X if present within the last 24 hours.

For the current study, only patients with a single DASA score for a calendar day were included in the analysis. Data from up to the first seven days of DASA scoring administration for each patient were included in the analysis. This time frame was selected because the average length of stay on the inpatient unit is less than a week. The population consisted of adult patients (18 years and above). All patients on the units during these periods were included. Some patients were admitted multiple times, and only the data from their first admission were used.

For the purposes of this study, agitation was defined as those acts (verbal or physical) that led to individuals requiring interventions (i.e., pharmacological, seclusion, or physical restraint). The pharmacy provided an audit of all medications used for acute agitation on both units within this period. The mental health nursing administrator provided information regarding seclusion and restraints that occurred on the unit within this period. The need for medication intervention was determined by psychiatric providers based on the perceived acuity of the situation. Seclusion and restraints were determined by nursing staff or psychiatric providers based on acuity as well.

An anonymous Likert-scale survey was also used to investigate mental health nursing perceptions of DASA scoring after the two-and-a-half-month period of administration. The survey consisted of five questions about how user-friendly the tool was and the nursing perception of the utility of the tool. The survey was distributed online, and nurses who were trained in DASA scoring voluntarily completed the form.

Statistical analyses were conducted according to a case-control design. Cases were defined as those patients who required agitation intervention during a calendar day. Controls were those who did not require intervention. Analyses were performed independently for each calendar day. Average DASA scores for cases were compared against average DASA scores for controls using a two-sample t-test design. Descriptive analyses were completed for the nursing survey.

## Results

**Figure 1:**
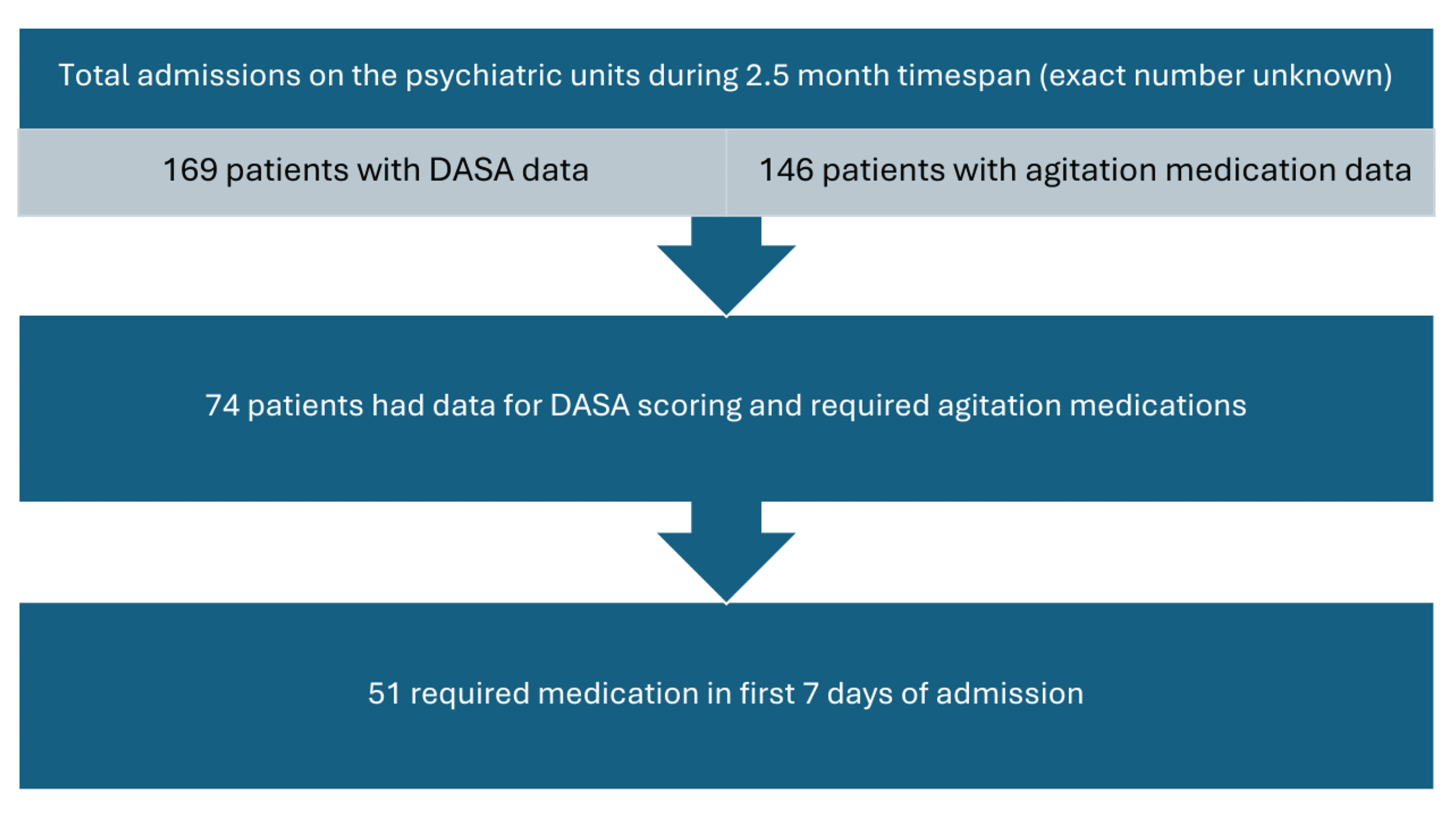
Data subgroups DASA: Dynamic Appraisal of Situational Aggression.

There were 169 patients with DASA scoring data. Demographic information is listed in Table 1. There were 146 patients with agitation medication data. Patients who lacked medication data did not require emergent medication for agitation within the first seven days of hospitalization. Seventy-four patients had both DASA score and agitation medication data. Fifty-one patients required medications within the first seven days of admission. A flowchart of these groups is shown in Figure 1. On day 1, there were 118 in the control group and 51 in the case group. The mean of the control group was 1, and the mean of the case group was 1.6, with a p value of 0.072 and an SD of 1.7-2.4. On day 2, there were 96 in the control group and 42 in the case group. The mean of the control group was 1.1, and the mean of the case group was 1.8, with a p value of 0.086 and an SD of 1.9-2.4. On day 3, there were 82 in the control group and 38 in the case group. The mean of the control group was 0.9, and the mean of the case group was 1.5, with a p value of 0.149 and an SD of 1.8-2.3. On day 4, there were 70 in the control group and 31 in the case group. The mean of the control group was 0.9, and the mean of the case group was 0.9, with a p value of 0.866 and an SD of 1.7-1.8. On day 5, there were 63 in the control group and 25 in the case group. The mean of the control group was 0.9, and the mean of the case group was 1.1, with a p value of 0.698 and an SD of 1.8-2.3. On day 6, there were 49 in the control group and 22 in the case group. The mean of the control group was 1, and the mean of the case group was 0.8, with a p value of 0.766 and an SD of 1.6-1.9. Finally, on day 7, there were 36 in the control group and 18 in the case group. The mean of the control group was 1.2, and the mean of the case group was 0.7, with a p value of 0.313 and an SD of 1.1-2. The results showed that there was no significant association between elevated DASA scores and the administration of emergent medications, seclusion, or restraint to manage agitation. As results were computed for each day following admission for seven days, none of the seven days showed elevated DASA scores in those patients who required agitation interventions compared with the group that did not require interventions (p>0.05 in all instances).

**Table 1:** Demographic information *Includes somatic symptom disorder, malingering, autism spectrum disorder, insomnia disorder, neurocognitive disorders, and unspecified mood disorder.

Variables	Values
Gender	
Male	47.33%
Female	49.11%
Transgender male	2.36%
Transgender female	1.18%
Age	
18-24	26.63%
25-34	20.11%
35-44	19.53%
45-54	11.83%
55-64	13.61%
65+	8.28%
Primary diagnosis	
Depressive disorders	21.89%
Psychotic disorders	22.49%
Bipolar-related disorders	9.47%
Trauma/stressor-related disorders	13.61%
Personality disorders	10.65%
Substance-related disorders	13.61%
Anxiety-related disorders	1.18%
Other*	7.10%

Results from an anonymous survey regarding DASA showed that among the 20 mental health nurses who responded, all of whom had completed training for DASA, 15% strongly agreed that the DASA scale impeded workflow, 25% agreed, 25% were undecided, 15% disagreed, and 20% strongly disagreed. When asked if the scoring scale was user-friendly, 5% strongly agreed, 85% agreed, 5% were undecided, none disagreed, and 5% strongly disagreed. In response to whether the DASA scale allowed nurses to better understand patients’ level of agitation, none strongly agreed, 5% agreed, 5% were undecided, 30% disagreed, and 60% strongly disagreed. When asked if nurses thought the tool would be helpful if linked to preventions or interventions for agitation, 5% strongly agreed, 25% agreed, 15% were undecided, 25% disagreed, and 30% strongly disagreed. When asked if nurses felt it was easier to communicate with staff about agitation using the DASA scale, the responses were 5% strongly agreed, 5% agreed, 5% were undecided, 20% disagreed, and 65% strongly disagreed (Table 2).

**Table 2:** Nursing survey results DASA: Dynamic Appraisal of Situational Aggression.

Survey question	Strongly agree	Agree	Undecided	Disagree	Strongly disagree
Did DASA impede workflow?	15%	25%	25%	15%	20%
Is DASA user-friendly?	5%	85%	5%	0%	5%
Did DASA allow a better understanding of patients’ level of agitation?	0%	5%	5%	30%	60%
Would DASA be helpful if linked to preventions or interventions for agitation?	5%	25%	15%	25%	30%
Was it easier to communicate with staff about agitation using DASA?	5%	5%	5%	20%	65%

## Discussion

The above results show no association between DASA scores completed by mental health nursing staff over a two-and-a-half-month span and associated interventions for agitation, such as medications to decrease the level of agitation, seclusion, or restraint. There are many dynamic factors that could be associated with this lack of association.

There may be a limitation regarding the type of aggression that can be predicted with this scoring tool. As seen in past data, impulsive aggression often occurs in inpatient units [3], which can be difficult to predict when there is a high turnover of patients, as seen on these inpatient psychiatric units, since it is an acute care facility. The average length of stay on the units studied was seven days. This tool is often used on forensic mental health units that may have a longer length of stay, which would allow for more assessment of the patient’s baseline and associated precipitating factors that increase the likelihood of agitation. The short length of stay limits factors in assessment.

The demographics of patients on inpatient psychiatric units may vary from those in other facilities, such as forensic facilities, and may require different factors to be considered in understanding their risk. It is appreciated, however, that this tool was validated in the ED and psychiatric acute stay inpatient unit settings in other studies.

With the high turnover in inpatient units, there is also constant flux in the dynamics of the milieu, which can lead to additional vulnerabilities. Changes in mental health nursing staff per shift can impact patients, as they may have built rapport with the previous staff member and may struggle with the transition, which could affect the prevalence of agitation. A previous study in a child and adolescent inpatient psychiatry setting showed that a dedicated milieu nurse (utilizing cognitive milieu therapy and nurse presence) had a significant impact on reducing the number of restraints when compared with the traditional patient-to-nurse assignment, with a monthly restraint rate of 72.9 per 1,000 client days during control versus 7.5 per 1,000 client days during intervention [12]. Seeing the impact of a unique nursing strategy on the number of restraints shows the potential impact of nursing staffing changes on the units studied in this paper. The change in staffing also leads to changes in the way scoring is completed, as each staff member may have worked with a specific patient for a limited time and may not understand their baseline. A prior nurse may report irritability, whereas the next nurse may not consider the same behavior as irritable, and this subjectivity can limit proper continuity in predicting aggression. The tool was scored once during a shift, which can make it difficult to create an overall average if one of the factors (i.e., impulsivity) was present or not present during the shift.

As seen in Table 1, there was a variety of primary diagnoses observed in the two-and-a-half-month time span. Agitation has the potential to be seen in any diagnosis, but factors such as irritability and impulsivity, which are assessed in DASA, may occur more often within the context of certain diagnoses. Though not analyzed in this study, exploring the likelihood of higher DASA scores or interventions within groups delineated by primary diagnosis may provide more information on predicting the likelihood of agitation events in this population. It may also provide insight into how agitation may be treated differently by providers based on primary diagnosis.

Another component to consider is the nurses’ perception of the scoring tool, as this can play a large role in the way the instrument is used. Results from the anonymous survey show that there was a range of perceptions regarding whether the scale impeded workflow, but the majority felt the tool was user-friendly. Most nurses who responded did not feel the scale allowed them to better understand the patients’ level of agitation or communicate about agitation with other staff members. There was variation in responses regarding whether the scale would be useful if associated with preventions or interventions. The perceived limited benefit from mental health nursing staff may impact motivation to complete scoring. One study researching barriers to implementation of the DASA scoring tool among 24 nurses listed multiple possible barriers impacting its use, such as preference for clinical intuition over the scale, concern that the scale would be hard to link with interventions, and limited time to familiarize themselves with past notes about the patient for baseline [13]. In a large metropolitan hospital in the upper Midwest of the United States, a scale was administered to nurses following DASA scoring for 13 weeks. Of these nurses, 66.7% believed DASA identified potential for patient violence, but only 48.7% endorsed continued use of DASA [14]. There could be a variety of reasons for this sentiment, but belief in the benefit of the tool can impact adherence and effectiveness.

In this study, when the scale was first implemented on the inpatient units, there were initial difficulties in compliance with daily scoring, which may also have impacted the results. As the nurses continued scoring, there was more consistent data. This is a limitation of the study. In addition, seclusion and restraint occurred less often than emergent medication intervention, and the limited occurrence of these events may have reduced the ability to detect significance. Future studies with longer time spans could be beneficial in determining the significance or lack thereof.

A study mentioned in the introductory section of this manuscript also showed a lack of validity of the scale, which could suggest that this tool is not predictive of agitation. However, given the factors listed above and the limitations of this study, a combination of factors could explain the lack of association.

## Conclusions

A significant association between DASA scores on the two inpatient units and interventions for acute agitation was not seen in this quality improvement project. There is a benefit in having more data on the implementation of this tool for mental health nursing in varied populations to assess the feasibility of using it across different units. This study assessed the implementation of the DASA tool in an upstate New York acute psychiatric care population, which is a demographic that has not been studied in past literature. The anonymous survey showed that the majority of nursing staff who responded found the tool user-friendly, though most did not think it would be helpful if linked to preventions or interventions. Evaluation of the potential factors contributing to the lack of significance also highlights the complex nature of this tool’s implementation. Aggression in inpatient psychiatric units remains a key area of study to expand upon in the years to come, given its impact on both patients and staff. Continued research on the use of tools to assess agitation will allow for further understanding to guide the prevention of agitation.
